# An E3 Ubiquitin Ligase RNF139 Serves as a Tumor-Suppressor in Glioma

**DOI:** 10.1007/s12031-021-01860-4

**Published:** 2021-06-09

**Authors:** Xiaofeng Chen, Weiping Kuang, Yong Zhu, Bin Zhou, Xiaosong Li, Xi Zhang, Bo Li, Liang Li, Shucheng Zou

**Affiliations:** 1 Department of Neurosurgery, Hunan Brain Hospital, Clinical Medical School of Hunan, University of Chinese Medicine, Changsha, 410007 Hunan Province China; 2 Provincial Key Laboratory of TCM Diagnostics, Hunan University of Chinese Medicine, Changsha, 410208 Hunan Province China

**Keywords:** Glioma, E3 ubiquitin ligase, RNF139, The PI3K/AKT signaling

## Abstract

Glioma is highly lethal because of its high malignancy. Ubiquitination, a type of ubiquitin-dependent protein modification, has been reported to play an oncogenic or tumor-suppressive role in glioma development, depending on the targets. Ring finger protein 139 (RNF139) is a membrane-bound E3 ubiquitin ligase serving as a tumor suppressor by ubiquitylation-dependently suppressing cell growth. Herein, we firstly confirmed the abnormal downregulation of RNF139 in glioma tissues and cell lines. In glioma cells, ectopic RNF139 overexpression could inhibit, whereas RNF139 knockdown could aggravate the aggressive behaviors of glioma cells, including hyperproliferation, migration, and invasion. Moreover, in two glioma cell lines, RNF139 overexpression inhibited, whereas RNF139 knockdown enhanced the phosphorylation of phosphatidylinositol 3-kinase (PI3K) and AKT serine/threonine kinase 1 (AKT). In a word, we demonstrate the aberration in RNF139 expression in glioma tissue samples and cell lines. RNF139 serves as a tumor-suppressor in glioma by inhibiting glioma cell proliferation, migration, and invasion and promoting glioma cell apoptosis through regulating PI3K/AKT signaling.

## Introduction

Glioma, the most common primary intracranial tumor accounting for more than 80% of craniocerebral malignant tumors, is highly lethal because of its high malignancy (Ostrom et al. 2014). During the past decades, genomic analysis of gliomas has provided new evidence for risk and prognosis. For example, critical events reported include the methylation of O^6^-methylguanine-DNA methyltransferase (Chen et al. 2013; Esteller et al. 2000), the mutation in isocitrate dehydrogenase (Brennan et al. 2013), the ubiquitination and degradation of key factors depending on ubiquitin (Ciechanover 2005; Muratani and Tansey 2003; Pan et al. 2015; Sang et al. 2019), and so on. These biomarkers might also be promising treatment targets.

Protein modification by ubiquitin, known as ubiquitination, could regulate proteasomal degradation, thus affecting multiple cellular processes, such as cell cycle regulation (Koepp et al. 1999), cellular response to inflammation (Ghosh et al. 1998), and antigen presentation (Rock and Goldberg 1999). As a result, the aberrations of ubiquitination, not surprisingly, might be associated with multiple pathologic states including cancer. Notably, either oncogene or suppressor gene products could be the targets of ubiquitination (Ciechanover et al. 1991, 1984; Kibel et al. 1995; Rogers et al. 1986); thus, deregulated proteasomal degradation, depending on the targets, could either play an oncogenic or tumor-suppressive role. In general, a three-tiered enzymatic cascade, including E1 (also known as ubiquitin-activating enzyme), E2 (also known as ubiquitin-conjugating enzyme), and E3 (also known as ubiquitin ligases), is necessary for tuning the whole ubiquitination (Pickart 2001; Zheng and Shabek 2017). Since the binding of proper E3 ubiquitin ligase to the target protein is an indispensable step for ubiquitination, E3 ubiquitin ligases are central functional modules during ubiquitination.

RNF139 (also known as TRC8) is a membrane-bound E3 ubiquitin ligase, which plays a tumor-suppressive role (Gemmill et al. 1998) by ubiquitylation-dependently suppressing cell growth (Brauweiler et al. 2007). For example, Gimelli et al. (Gimelli et al. 2009) demonstrated the disruption of RNF139 in a young girl with dysgerminoma. Regarding the tumor-suppressive functions, Brauweiler et al. (Brauweiler et al. 2007) reported RNF139-caused inhibition of human kidney cell proliferation by inducing cell cycle arrest in G2/M phase, inhibiting DNA synthesis, and promoting apoptosis. Moreover, in a RING-dependent manner, RNF139 suppressed the tumor formation in a nude mouse model (Brauweiler et al. 2007). Lin et al. (2013) reported that RNF139 led to the ubiquitination and degradation of heme oxygenase-1 (HO-1), an antioxidant enzyme overexpressed in multiple cancers. In tongue cancer cells, RNF139 silencing dramatically enhanced cancer cell invasion and enhanced SCC25 cell tumorigenicity in nude mice (Wang et al. 2017). Considering the tumor-suppressive role of RNF139 in several cancers, we hypothesize that RNF139 also plays a tumor-suppressive role against glioma.

In the present study, firstly, we confirmed the mRNA expression and protein levels of RNF139 in tissue samples and cell lines. Secondly, RNF139 overexpression and knockdown was achieved in glioma cell lines and the specific effects of RNF139 overexpression or knockdown on cancer cell proliferation, apoptosis, migration, and invasion were investigated. Finally, considering that the occurrence of glioblastoma multiform, the most malignant glioma, is highly relevant with changes in the factors of the epidermal growth factor receptor (EGFR) and PI3K/Akt/mechanistic target of rapamycin kinase (mTOR) signaling pathways (Chakravarti et al. 2004; Li et al. 2016), we investigated the alterations in the PI3K/AKT signaling in response to RNF139 overexpression or knockdown. Altogether, in the current study, for the first time, we tried to reveal the biological functions and the potential molecular mechanism of RNF139 in glioma. We observed that the tumor-suppressive role of RNF139 in glioma cells through regulating the possible downstream signaling (PI3K/AKT signaling). Our basic experimental outcomes provided sufficient results to uncover a newfound mRNA/signaling pathway regulatory network of RNF139/PI3K/AKT signaling in glioma, supplying a crucial perception concerning the regulatory theory in glioma advancement and new therapeutic options of glioma diseases.

## Materials and Methods

### Clinical Samplin

Twelve tissue samples from the tumor core region and twelve samples from the peritumoral brain edema (PTBE) region were obtained from patients received treatment (surgical resection) at Hunan Brain Hospital. The sampling procedure was conducted with the approval of the Ethic Committee of Hunan Brain Hospital. Immediately after specimen sampling, tissues were transferred to formalin or − 80 °C container until subsequent experiments.

### Cell Lineage and Cell Culture

Normal human brain astroglia cell line SVG p12 (CRL-8621) was obtained from ATCC (Manassas, VA, USA) and cultured in EMEM (Gibco, Waltham, MA, USA). Normal human astrocyte (HA; Catalog #1800) was obtained from ScienCell (Carlsbad, CA, USA) and cultured in Astrocyte medium (AM; Catalog #1801, ScienCell). Glioma cell line U87 (HTB-14) was obtained from ATCC and cultured in EMEM (Gibco). Glioma cell line SHG-44 (3131C0001000700048) was obtained from Cell Resource Center, Shanghai Institute of Life Sciences, Chinese Academy of Sciences (Shanghai, China) and cultured in RPMI 1640 (w/o Hepes; Gibco). Glioma cell line GOS-3 (ACC 408) was obtained from German Collection of Microorganisms and Cell Cultures (Braunschweig, Germany) and cultured in Dulbecco’s MEM (Gibco). Glioma cell line TJ905 (3111C0001CCC000267) was obtained from Cell Resource Center, Shanghai Institute of Life Sciences, Chinese Academy of Sciences and cultured in DMEM-H: Dulbecco’s modified Eagle’s medium (DME H-21 4.5 g/Liter Glucose; Gibco). All glioma cell lines were cultured in medium supplemented with 10% FBS (Invitrogen, Waltham, MA, USA). All cells were cultured at 37 °C in 5% CO_2_.

### Cell Transfection

For RNF139 overexpression, full-length RNF139 gene was combined in pLVX-puro (RNF139) and the empty vector was used as a negative control (vector-NC). For RNF139 knockdown, short-hairpin RNA against RNF139 gene, as well as their nontargeting sequences were reconstructed in pLVX-shRNA2-puro (shRNA-NC, shRNA1-RNF139, shRNA2-RNF139; YouBio, China). The primer sequence for shRNA are listed in Table 1. Cells were seeded in a 24-well plate, and the Lipofectamine 3000 reagent (Thermo Fisher Scientific, Waltham, MA, USA) was used to transfect cells with the plasmids mentioned above. Cells were collected 48 h after transfection.

**Table 1:** ShRNA sequence of RNF139

Gene	Sequence (5′–3′)
shRNA1	
Top strand	CACCGCCTTTCTGTTAGCTGCAACTCGAAAGTTGCAGCTAACAGAAAGGC
Bottom strand	AAAAGCCTTTCTGTTAGCTGCAACTTTCGAGTTGCAGCTAACAGAAAGGC
shRNA2	
Top strand	CACCGCACTTTGCCTTCGGAAATGGCGAACCATTTCCGAAGGCAAAGTGC
Bottom strand	AAAAGCACTTTGCCTTCGGAAATGGTTCGCCATTTCCGAAGGCAAAGTGC
shRNA-NC	
Top strand	CACCGCCTTTCTGTTAGCTGCAACTCGAAAGTTGCAGCTAACAGAAAGGC
Bottom strand	CGGAAAGACAATCGACGTTGAGCTTTCAACGTCGATTGTCTTTCCGAAAA

### qRT-PCR

TRIzol reagent (Invitrogen) was used to extract total RNA from target cells or tissues, and then use the first strand cDNA synthesis kit (Promega) for reverse transcription. A SYBR Green PCR Master Mix (Qiagen) was used to detect the expression level according to the manufacturer’s instructions. With GAPDH expression as an internal reference, calculate the relative expression using the Ct method. The specific primers used are presented in Table 2.

**Table 2:** qRT-PCR primer sequences

Primer	Sequence (5′–3′)
RNF139	
Forward primer	TACCCGGATTCCAGCCAAAG
Reverse primer	GGGACCTTTTCGAGGAAGCA
GAPDH	
Forward primer	AGCCACATCGCTCAGACAC
Reverse primer	GCCCAATACGACCAAATCC

### Immunoblotting

Extract total protein from target cells, separate the total protein samples using SDS-PAGE, and transfer the protein samples to polyvinylidene fluoride membranes. For the blockage of nonspecific binding, the membranes were incubated with 5% skim milk dissolved in diphenyltris buffer saline for 2 h at room temperature. Incubate the membranes with the primary antibodies against RNF139 (Catalog # MBS421811; MyBioSource, Inc., San Diego, CA, USA), PI3K (AF6241; Affinity, Cincinnati, OH), p-PI3K (AF3242, Affinity), Akt (Y409094; ABM, Richmond, Canada), or p-Akt (Y011054, ABM) overnight at 4 °C. The incubation with the primary antibodies was followed by another incubation with the corresponding horseradish peroxidase coupled secondary antibody for 2 h at room temperature. Finally, the enhanced chemiluminescence visualization scan was performed.

### MTT Assay for Cell Viability

Target cells were transfected and seeded in 96-well plates at a density of 1 × 10^4^ cells/well for a 24, 48, or 72 h of incubation. At the end of each incubation, 10 μl of 5 mg/ml MTT (added to PBS; Sigma-Aldrich, St. Louis, MO, USA) was added to each well and the cells were subjected to another 4 h of incubation. Remove the supernatants, add 100 μl dimethyl sulfoxide (DMSO, Thermo Fisher Scientific, Waltham, MA, USA) to each well, measure the absorbance value at 490 nm using a microplate reader.

### EdU Assay for DNA Synthesis

Use 5-ethyl-2'-deoxyuridine (EdU) detection kit (EdU kit; Ribobio, Guangzhou, China) for determining DNA synthesis. Cells were firstly transfected, incubated with EdU for 2 h, and collected. Then, fix the cells in 4% formaldehyde for 20 min and permeabilize the cells. Wash the cells with PBS, treat the cells with 200 μl Apollo ® reaction cocktail (Ribobio) for 10 min, and permeabilize the cells. Stain the cells with 100 μl Hoechst 33,342 (5 μg/ml; Thermo Fisher Scientific). Observe the cells under a fluorescence microscope (Olympus, Tokyo, Japan).

### Flow Cytometry for Cell Apoptosis

Target cells were transfected and examined for cell apoptosis using a FITC-labeled AnnexinV/propidium iodide (PI) Apoptosis Detection kit (Beyotime, Shanghai, China). Immediately after staining, flow cytometry was performed (Beckman, USA). Early apoptotic cells were positive for Annexin V, and late apoptotic cells were positive for both Annexin V and PI.

### Transwell for Cell Migration and Invasion

Target cells were transfected and evaluated for the invasion using Matrigel Transwell (Corning BioCoat Matrigel Invasion Chambers; Corning, Corning, NY, USA). The cells were seeded in a 6-well plate for 12 h at a density of 5 × 10^5^ cells/well. Subsequently, the cells were cultured with serum-free medium for 12 h and then seeded in 100 μl serum-free media into each well of the Transwell at a density of 2 × 10^4^ cells/well. In the lower chamber, add 700 μl complete medium containing 10% FBS. After 48 h, remove the cells stayed on the upper chambers, fix the cells on the surface of the lower chamber with 4% paraformaldehyde, and stain these cells with crystal violet. Count the number of cells on the surface of the lower chamber (invaded cells). Cell migration was evaluated similarly except for using Transwell without Matrigel.

### Statistics Analysis

Each experiment was repeated for at least three times. Data were processed using the GraphPad software (San Diego, CA, USA) and shown as mean ± standard deviation (SD). A one-way analysis of variance (ANOVA) and then Tukey’s multiple comparison test or Student’s *t* test was performed to analyze the statistical significance. The cell experiments were performed at least three times. A *P* value of < 0.05 was considered statistically significant.

## Results

### The Downregulation of RNF139 in Glioma Tissues and Cell Lines

**Fig. 1:**
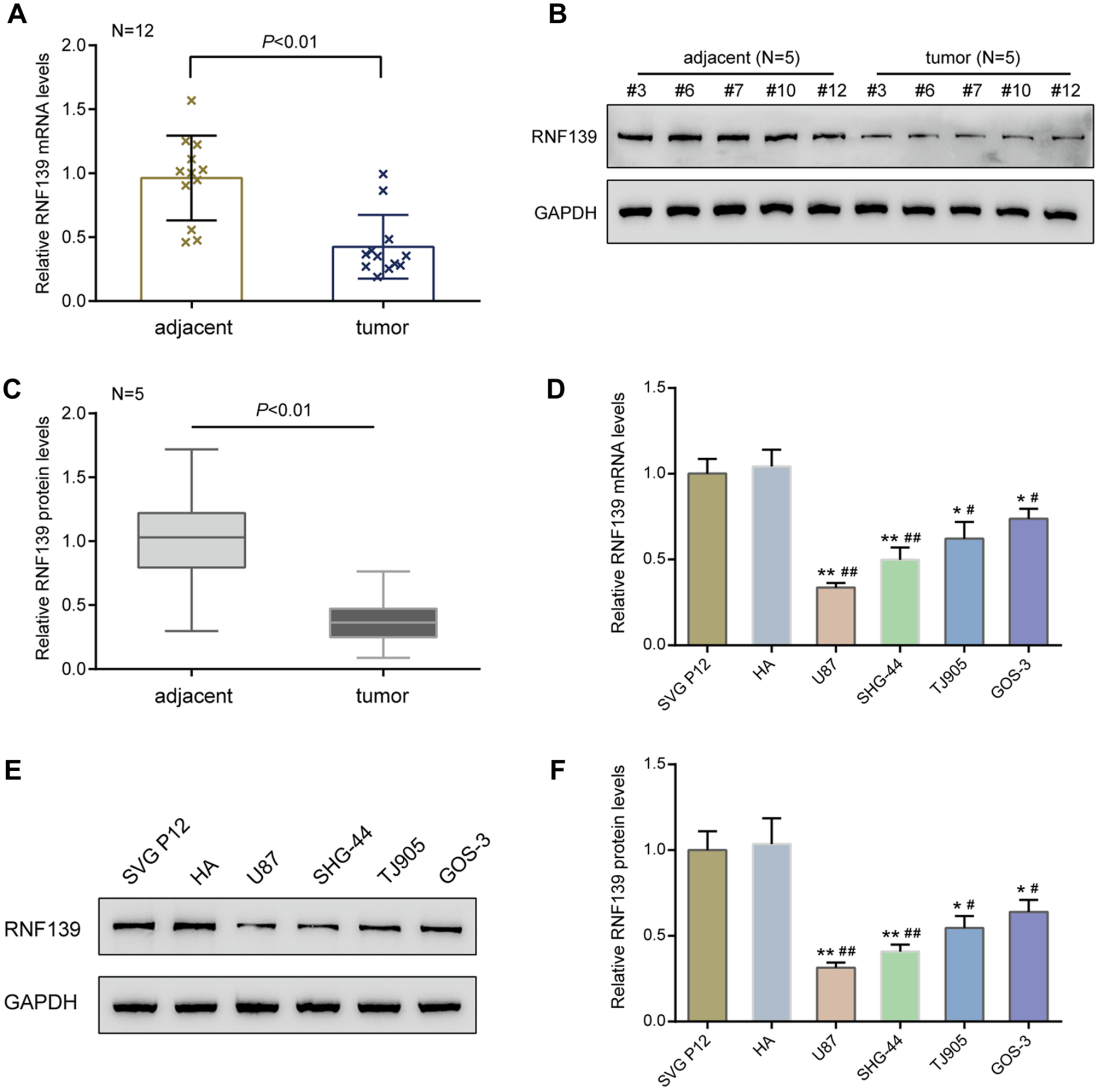
The downregulation of RNF139 in glioma tissues and cell lines. **A** The expression of RNF139 was examined in 12 paired glioma and adjacent non-cancerous tissues by qRT-PCR. *N* = 12. **B**, **C** The protein levels of RNF139 were examined in tissue samples by immunoblotting. *N* = 5. **D** The expression of RNF139 was examined in human astrocyte cell lines, SVG P12 and HA, and glioma cell lines, U87, SHG-44, GOS-3, TJ905 by qRT-PCR. *N* = 3. **E**, **F** The protein level of RNF139 was examined in human astrocyte cell lines, SVG P12 and HA, and glioma cell lines, U87, SHG-44, GOS-3, TJ905 by immunoblotting. *N* = 3, *P < 0.05, **P < 0.01 compared to SVG P12 group; #*P* < 0.05, ##*P* < 0.01 compared to HA group

Before investigating the specific effects of RNF139 on glioma cell phenotypes, we firstly verified tissue RNF139 expression in glioma and adjacent noncancerous specimens; as shown in Fig. 1A, RNF139 mRNA levels were remarkably downregulated in tumor tissues compared with the control group (*N* = 12). Consistently, the protein levels of RNF139 were significantly decreased in tumor tissues compared with those in adjacent non-cancerous tissues (*N* = 5) (Fig. 1B, C). In cell lines, the expression of RNF139 was dramatically downregulated in glioma cell lines, including U87, SHG-44, GOS-3, and TJ905 compared with that in human astrocyte cell lines, SVG P12 and HA (Fig. 1D). Similarly, the protein levels of RNF139 were significantly decreased in glioma cell lines, including U87, SHG-44, GOS-3, and TJ905 compared with those in human astrocyte cell lines, SVG P12 and HA (Fig. 1E, F). These findings confirm the abnormal downregulation of RNF135 in glioma.

### Effects of RNF139 Overexpression and Knockdown on Glioma Cell Phenotypes

**Fig. 2:**
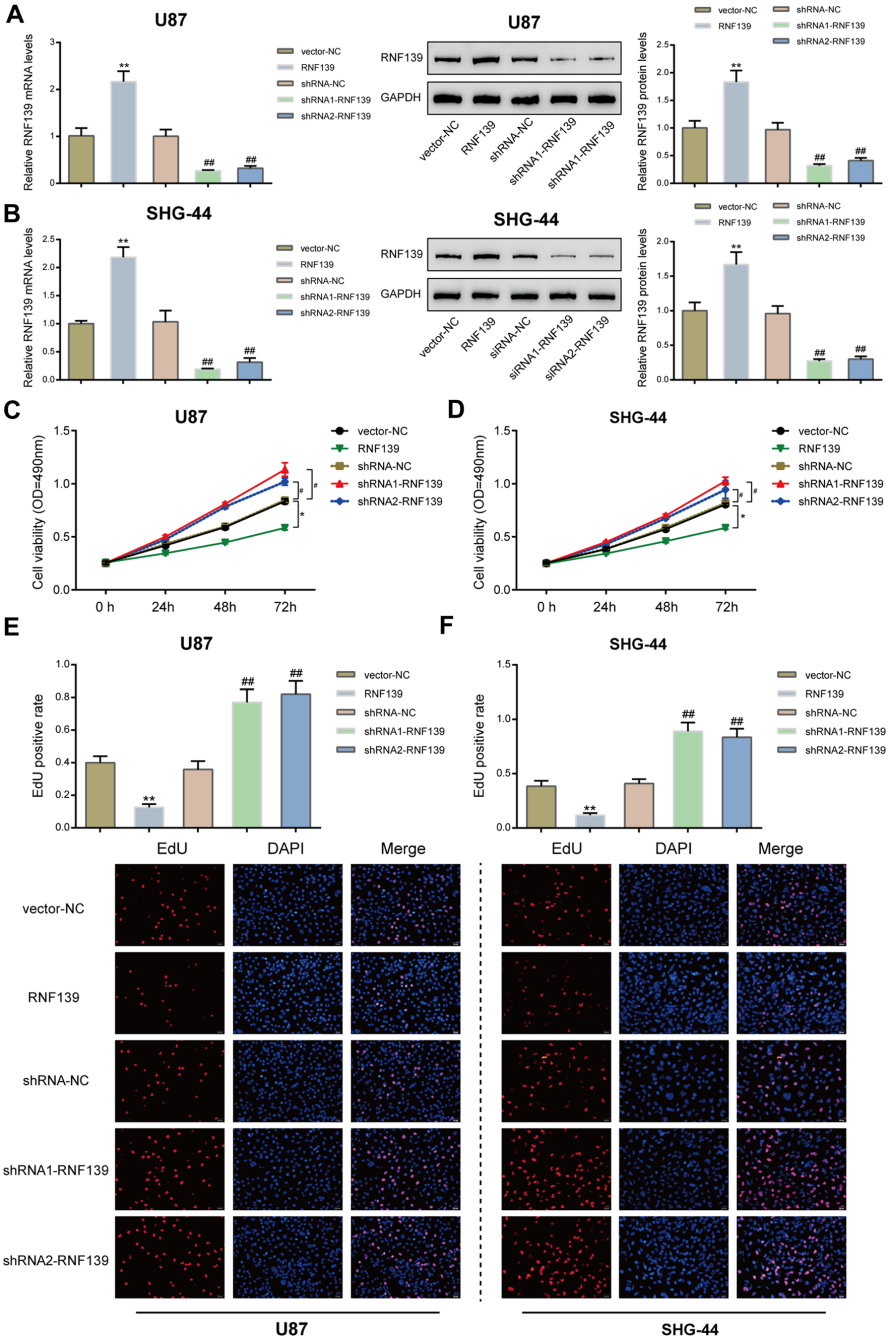
Effects of RNF139 overexpression and knockdown on glioma cell proliferation. **A**, **B** RNF139 overexpression or knockdown was achieved in U87 and SHG-44 cells by transfecting RNF139-overexpressing vector (RNF139) or small interfering RNA targeting RNF139 (shRNA1-RNF139 and shRNA2-RNF139). The expression levels of RNF139 were confirmed by qRT-PCR and immunoblotting. Next, U87 and SHG-44 cells were transfected with RNF139 or shRNA1-RNF139/shRNA2-RNF139 and examined for cell viability by MTT assay (**C**, **D**) and DNA synthesis by EdU assay (**E**, **F**). *N* = 3, *P < 0.05, **P < 0.01 compared to vector-NC group; #*P* < 0.05, ##*P* < 0.01 compared to shRNA-NC group

**Fig. 3:**
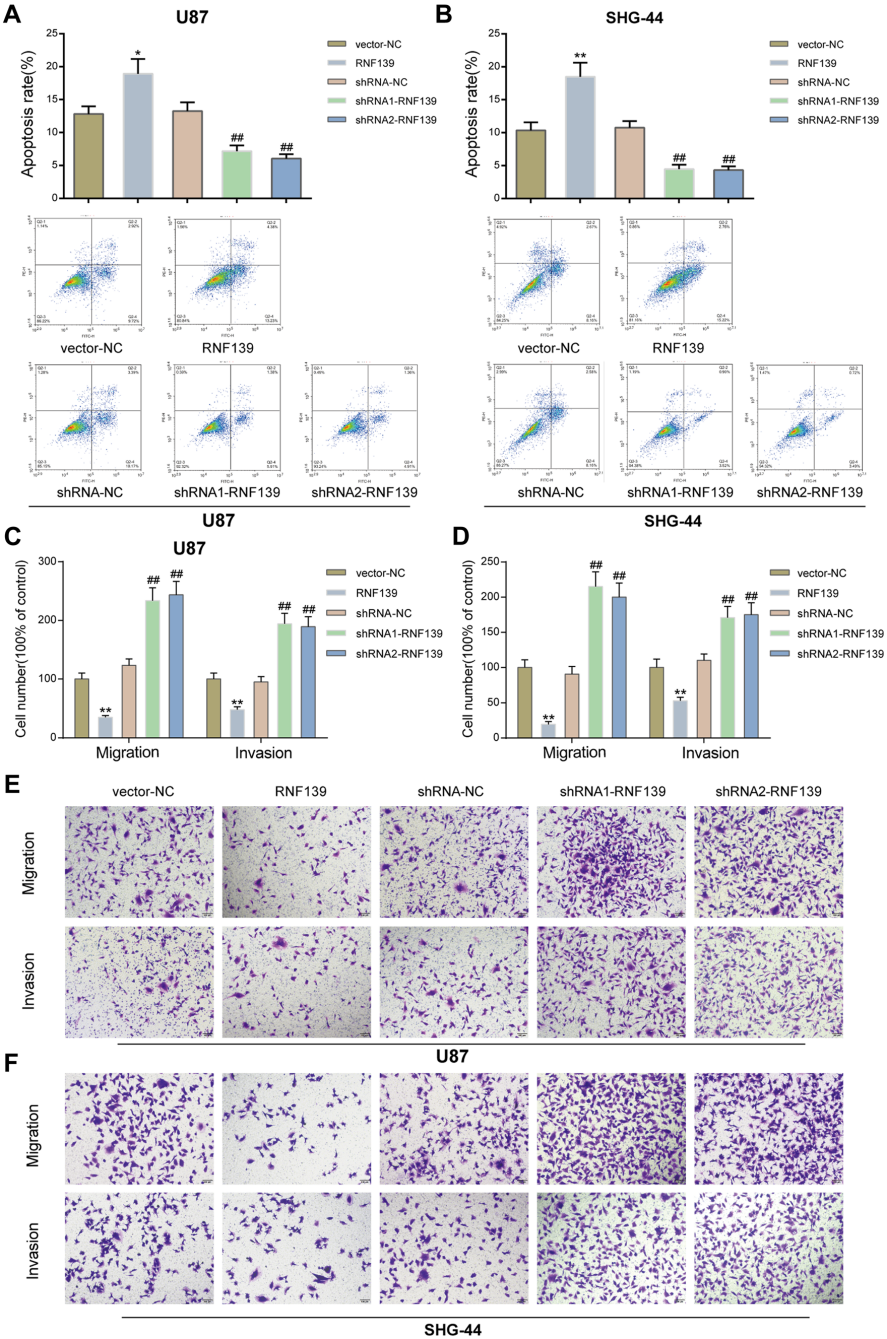
Effects of RNF139 overexpression and knockdown on glioma cell apoptosis, migration, and invasion. U87 and SHG-44 cells were transfected with RNF139 or shRNA1-RNF139/shRNA2-RNF139 and examined for cell apoptosis by flow cytometry (**A**, **B**) and cell migration and invasion by Transwell assay (**C**–**F**). *N* = 3, *P < 0.05 compared to vector-NC group; **P < 0.01, ##*P* < 0.01 compared to shRNA-NC group

The abnormal downregulation of RNF139 suggests that it may serve as a tumor-suppressor against glioma. For verifying the speculation, we achieved RNF139 overexpression or knockdown in U87 and SHG-44 cells by transfecting RNF139-overexpressing vector (RNF139) or small interfering RNA targeting RNF139 (shRNA1-RNF139 and shRNA2-RNF139). The mRNA expression and protein levels of RNF139 were confirmed by qRT-PCR and Immunoblotting (Fig. 2A, B); RNF139 overexpression or knockdown was successfully conducted. Next, U87 and SHG-44 cells were transfected with RNF139 or shRNA1-RNF139/shRNA2-RNF139 and examined for cell viability, DNA synthesis, cell apoptosis, cell migration, and cell invasion. In both glioma cell lines, RNF139 overexpression significantly inhibited tumor cell viability (Fig. 2C, D), DNA synthesis (Fig. 2E, F), and cell migration and invasion (Fig. 3C–F), whereas promoted cell apoptosis (Fig. 3A, B). By contrast, when RNF139 was knocked down in glioma cell lines, cell viability (Fig. 2C, D), DNA synthesis (Fig. 2E, F), and cell migration and invasion (Fig. 3C–F) were all enhanced, whereas cell apoptosis was inhibited (Fig. 3A, B). These data indicate that ectopic RNF139 overexpression could inhibit, whereas RNF139 knockdown could aggravate the aggressive behaviors of glioma cells.

### RNF139 Could Affect the Activation of the PI3K/AKT Signaling Pathway

**Fig. 4:**
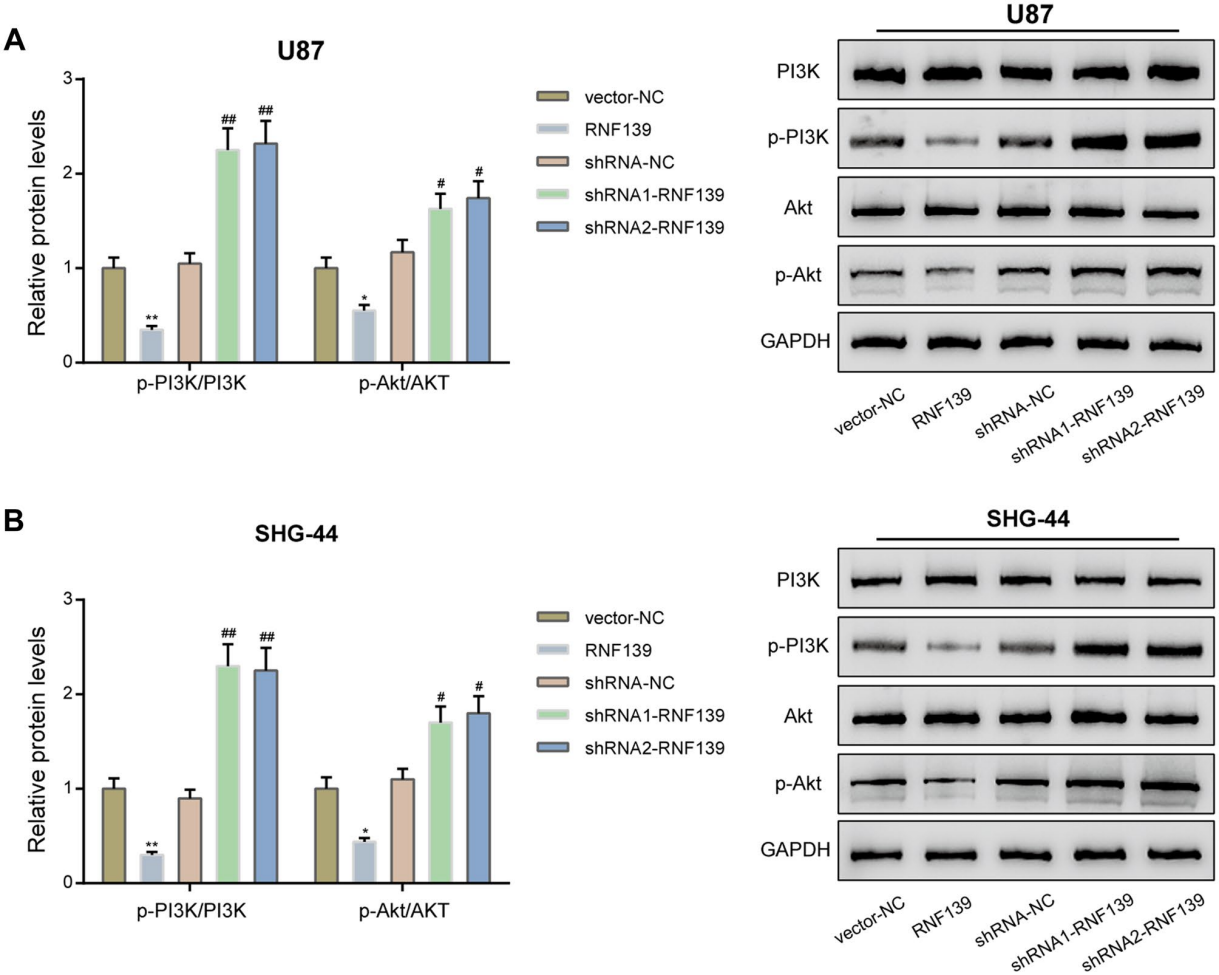
The PI3K/AKT signaling pathway is involved in the effects of RNF139. U87 and SHG-44 cells were transfected with RNF139 or shRNA1-RNF139/shRNA2-RNF139 and examined for the protein levels of PI3K, p-PI3K, Akt, and p-Akt by immunoblotting (**A**, **B**). *N* = 3, *P < 0.05, **P < 0.01 compared to vector-NC group; #*P* < 0.05, ##*P* < 0.01 compared to shRNA-NC group

The aggressiveness of cancer cells could result from the deregulation of complex alternate signaling pathways (Alifieris and Trafalis 2015); among the deregulated signaling pathways, the PI3K/AKT signaling showed to be a crucial one in glioma pathogenesis (Tong et al. 2016; Wen and Kesari 2008; Zhao et al. 2017). Therefore, we investigated whether the PI3K/AKT signaling pathway could also be affected by RNF139 in glioma cells. U87 and SHG-44 cells were transfected with RNF139 or shRNA1-RNF139/shRNA2-RNF139 and examined for the protein levels of PI3K, p-PI3K, Akt, and p-Akt. As shown in Fig. 4A, B, in both glioma cell lines, RNF139 overexpression dramatically decreased the ratio of p-PI3K/PI3K and p-AKT/AKT; on the contrary, RNF139 knockdown induced by either shRNA1-RNF139 or shRNA2-RNF139 elevated the ratio of p-PI3K/PI3K and p-AKT/AKT. Thus, RNF139 overexpression could inhibit the hyper-activation of the PI3K/AKT signaling pathway in glioma cells.

## Discussion

In the present study, we firstly confirmed the abnormal downregulation of RNF139 in glioma tissues and cell lines. In glioma cells, ectopic RNF139 overexpression could inhibit, whereas RNF139 knockdown could aggravate the aggressive behaviors of glioma cells, including hyperproliferation, migration, and invasion. Moreover, in two glioma cell lines, for the first time, RNF139 was identified that played a tumor suppressor role through regulating PI3K/AKT signaling. RNF139 overexpression inhibited, whereas RNF139 knockdown enhanced the phosphorylation of PI3K and AKT.

Similar as phosphorylation, ubiquitination is another most prevalent and critical protein modifications occurring inside the cell. Since ubiquitination could occur in a wide range of proteins, ubiquitination participates in cellular processes including cell cycle progression, cell apoptosis, receptor downregulation, and gene transcription. Thus, not surprisingly, ubiquitination has been described in multiple cancer-related pathways. During ubiquitination, a ubiquitin is connected to the target protein by a covalent isopeptide bond between ubiquitin C-terminal and target protein lysine residue. Because of its role in interacting with substrates and determining the substrate specificity, the E3 ubiquitin ligases are crucial in the ubiquitin-conjugation system. Not surprisingly, the functions of E3 ligases have been reported in cancers, either by dictating the degradation of oncogenic proteins or tumor-suppressive proteins. As we have mentioned, FBW7 agonist leads to the degradation of c-MYC and cyclin E in cancer cells (Diefenbacher et al. 2015). Another group demonstrated that nuclear TRIM59, as an E3 ligase, led to the ubiquitination of mH2A1 and affected mH2A1 stability in glioblastoma (Sang et al. 2019). Similarly, stable SHPRH, as an E3 ligase, ubiquitinated proliferating cell nuclear antigen (PCNA), thus inhibiting glioma cell proliferation and tumorigenicity (Zhang et al. 2018). On the contrary, the ubiquitin-protein ligase E3C (UBE3C) led to the ubiquitination and degrading of Annexin A7, promoting glioma development (Pan et al. 2015). In the present study, we firstly found the abnormal downregulation of RNF139, an E3 ubiquitin ligase, in glioma tissue samples and cell lines. The downregulation of RNF139 in glioma suggests that RNF139 might play a role in glioma development.

Interestingly, RNF139 has been previously regarded as a tumor-suppressive factor in several cancers, including tongue cancer (Wang et al. 2017), renal cancer (Brauweiler et al. 2007), kidney carcinoma A498 cells, osteosarcoma U2OS cells, and cervical carcinoma HeLa cells (Lin et al. 2013). In these cancer cells, ectopic RNF139 expression has been reported to induce cell cycle arrest, suppress cancer cell proliferation, migration and invasion, and inhibit the tumor formation in nude mice. Glioma is characterized by rapid cell proliferation and angiogenesis (Peng et al. 2018). Besides, similar to the spreading process of metastasis, single glioma cells could be observed in the far side of the brain (Kirsch et al. 2000). Thus, to investigate the specific role of RNF139 in glioma carcinogenesis, we conducted ectopic expression or knockdown of RNF139 in glioma cells and examined the cell viability, DNA synthesis, cell apoptosis, migration, and invasion. Consistent with its abnormal downregulation in glioma, ectopic RNF139 expression significantly suppressed glioma cell proliferation, migration and invasion, and promoted cell apoptosis; RNF139 knockdown exerted opposite effects on glioma cell phenotypes. These findings strongly support the tumor-suppressive role of RNF139 in glioma.

Genetic aberrations have been frequently found in glioblastoma, one of the most malignant gliomas. These aberrations include EGFR, platelet-derived growth factor receptor alpha (PDGFRA), phosphatidylinositol-4,5-bisphosphate 3-kinase catalytic subunit alpha (PIK3CA), phosphatase and tensin homolog (PTEN), tumor protein p53 (TP53), cyclin-dependent kinase inhibitor 2A/B (CDKN2A/B), and so on, leading to dysfunction of the PI3K/Akt/mTOR, p53, RB transcriptional corepressor 1 (RB1), and other signaling pathways; however, the discovery of these aberrations also open up targeted therapies through targeting hyper-activated pathways [4]. The crucial role of the PI3K/Akt signaling pathway has been widely recognized in mediating cell proliferation, apoptosis, motility, as well as angiogenesis in glioblastoma (Brennan et al. 2013). Due to the cellular functions, elevated PI3K and AKT are poor prognostic factor in patients with malignant gliomas (Chakravarti et al. 2004). In the present study, we also monitored the alterations in the PI3K/AKT signaling in response to RNF139 overexpression or knockdown. Consistent with previous studies, RNF139 overexpression inhibited, whereas RNF139 knockdown enhanced the phosphorylation of PI3K and AKT. These findings suggest that the PI3K/AKT signaling might mediate the tumor-suppressive role of RNF139 in glioma for the first time.

Altogether, we demonstrate the aberration in RNF139 expression in glioma tissue samples and cell lines. RNF139 serves as a tumor-suppressor in glioma by inhibiting cancer cell proliferation, migration, and invasion and promoting cancer cell apoptosis through regulating PI3K/AKT signaling.

## Data Availability

Please contact the authors for data requests.

## Abbreviations

AKT: AKT serine/threonine kinase 1
CDKN2A/B: Cyclin-dependent kinase inhibitor 2A/B
EdU: 5-Ethyl-2'-deoxyuridine
EGFR: Epidermal growth factor receptor
HO-1: Heme oxygenase-1
mTOR: Mechanistic target of rapamycin kinase
PCNA: Proliferating cell nuclear antigen
PDGFRA: Platelet-derived growth factor receptor alpha
PI: Propidium iodide
PIK3CA: Phosphatidylinositol-4,5-bisphosphate 3-kinase catalytic subunit alpha
PI3K: Phosphatidylinositol 3-kinase
PTBE: Peritumoral brain edema
PTEN: Phosphatase and tensin homolog
RB1: RB transcriptional corepressor 1
RNF139: Ring finger protein 139
TP53: Tumor protein p53
UBE3C: Ubiquitin-protein ligase E3C
